# Diacylglycerol kinase η colocalizes and interacts with apoptosis signal-regulating kinase 3 in response to osmotic shock

**DOI:** 10.1016/j.bbrep.2021.101006

**Published:** 2021-04-27

**Authors:** Yuji Suzuki, Maho Asami, Daisuke Takahashi, Fumio Sakane

**Affiliations:** a Department of Chemistry, Graduate School of Science, Chiba University, Chiba 263-8522, Japan; b Department of Pharmaceutical Health Care and Sciences, Kyushu University, Fukuoka 812-8582, Japan

**Keywords:** Diacylglycerol kinase, Apoptosis signal-regulating kinase, Osmotic shock, Stress granule, C-Raf

## Abstract

Diacylglycerol kinase (DGK) η translocates from the cytoplasm to punctate vehicles via osmotic shock. Apoptosis signal-regulating kinase (ASK) 3 (MAP kinase kinase kinase (MAPKKK) 15) is also reported to respond to osmotic shock. Therefore, in the present study, we examined the subcellular localization of DGKη and ASK3 expressed in COS-7 cells under osmotic stress. We found that DGKη was almost completely colocalized with ASK3 in punctate structures in response to osmotic shock. In contrast, DGKδ, which is closely related to DGKη structurally, was not colocalized with ASK3, and DGKη failed to colocalize with another MAPKKK, C-Raf, even under osmotic stress. The structures in which DGKη and ASK3 localized were not stained with stress granule makers. Notably, DGKη strongly interacted with ASK3 in an osmotic shock-dependent manner. These results indicate that DGKη and ASK3 undergo osmotic shock-dependent colocalization and associate with each other in specialized structures.

## Highlights

• DGKη translocates from the cytoplasm to punctate vehicles via osmotic stress.
• DGKη colocalizes with ASK3 in punctate vehicles in response to osmotic shock.
• DGKη interacts with ASK3 in response to osmotic shock.
• The punctate vesicles are unique and specialized for DGKη and ASK3.

## Abbreviations:

ASK: apoptosis signal-regulating kinase
BPD: bipolar disorder
DGK: diacylglycerol kinase
EGF: epidermal growth factor
ERK: extracellular signal-regulated kinase
G3BP1: Ras GTPase-activating protein SH3-domain-binding protein
KO: knockout
MAPK: mitogen-activated kinase
MAPKKK: MAPK kinase kinase
MEK: MAPK/ERK kinase
TIAR: T-cell intracellular antigen 1 related protein

## 1 Introduction

Diacylglycerol kinase (DGK) converts diacylglycerol to phosphatidic acid [[1], [2], [3], [4], [5]]. Both diacylglycerol [[6], [7], [8]] and phosphatidic acid [[9], [10], [11]] are known to serve as second messengers controlling many key enzymes. Therefore, DGK plays a critical role in the regulation of a wide variety of physiological and pathological phenomena.

DGK consists of ten isozymes (α, β, γ, δ, ε, ζ, η, θ, ι, κ) [[1], [2], [3], [4], [5]]. These DGK isozymes are divided into five groups (type I – V) according to their structural characteristics. DGKη belongs to the type II DGK group, which also contains DGKδ and κ [12,13]. Moreover, splice variants of DGKη exist, *i.e.*, DGKη1 – 4 [[13], [14], [15]]. DGKη1, which is a main alternative splicing product of the DGKη gene [13,16], has a pleckstrin homology domain at its N-terminal and a catalytic domain that is divided into two subdomains.

Several genome-wide association studies showed that the DGKη gene is associated with the etiology of bipolar disorder (BPD) [[17], [18], [19]]. Moreover, the DGKη gene is located within the BPD linkage region on 13q14 [20,21]. Notably, we recently generated DGKη-knockout (KO) mice and analyzed their phenotypes. Intriguingly, the DGKη-KO mice showed lithium-sensitive BPD (mania)-like behaviors [22,23]. Moreover we recently demonstrated that DGKη deficiency caused hyperactivity of the dopaminergic system [24]. Therefore, DGKη is attracting much attention as a crucial BPD-associated gene [25]. In addition, we reported that DGKη interacted with C-Raf and B-Raf (mitogen-activated kinase (MAPK) kinase kinase (MAPKKK)) in response to epidermal growth factor (EGF) stimulation and regulated the Raf–MAPK/extracellular signal-regulated kinase (ERK) kinase (MEK)–ERK pathway [26]. On the other hand, stress stimulation (osmotic shock) induced translocation of DGKη to nonionic detergent-resistant membranes [27] in punctate structures [13,28]. However, the exact nature of these punctate structures remains unclear.

Apoptosis signal-regulating kinase (ASK) 1 was identified as a MAPKKK that activates the MKK4/7-JNK and the MKK3/6-p38 pathways resulting in apoptosis [29]. ASK2 and ASK3 were subsequently found as members of the ASK family [30,31]. The ASK family was shown to be involved in a wide range of stress signaling pathways (oxidative, osmotic and endoplasmic reticulum stresses) and several diseases including cancer [32,33]. ASK3 (also known as MAPKKK15) is known to respond to osmotic stress and regulate p38 activity and is essential to regulate bidirectional cell volume under both hypoosmotic and hypoosmotic stresses [[34], [35], [36]].

In the present study, we examined the subcellular localization of DGKη1 and ASK3, and their interaction in response to osmotic shock. Intriguingly, we found that DGKη1 and ASK3 strongly interacted with one another, and moreover, were almost completely colocalized in punctate structures specialized for these enzymes in an osmotic stress-dependent manner.

## 2 Materials and methods

### 2.1 cDNA constructs

pAcGFP-C1-human DGKη1 [27] and p3 × FLAG-CMV-DGKη1 [13] were generated as described previously. cDNA encoding human DGKη1 was excised from pAcGFP-C1-human DGKη1 with SalI and BamHI and inserted into the SalI/BamHI site of the pDsRed-monomer-C1 vector (Takara-Clontech, Kusatsu, Japan). cDNA encoding human DGKδ2 was excised from pAcGFP-C1-human DGKδ2 [37] with EcoRI and SalI and inserted into the EcoRI/SalI site of the pDsRed-monomer-C1 vector. EGFP-tagged human ASK3 was generated as described previously [36].

### 2.2 Cell culture and transfection

COS-7 cells were maintained in Dulbecco's modified Eagle's medium (DMEM) (Wako Pure Chemical Industries, Osaka, Japan) supplemented with 10% fetal bovine serum (Biological Industries (Beit-Haemek, Israel)), 100 units/ml penicillin, and 100 μg/ml streptomycin (Wako Pure Chemical Industries) at 37 °C in an atmosphere containing 5% CO_2_. Cells were transiently transfected using PolyFect reagent (Qiagen, Hilden, Germany) as described by the manufacturer.

### 2.3 Confocal microscopy

After 24 h of transfection with DsRed-monomer-DGKη1 and/or EGFP-ASK3, COS-7 cells were incubated in DMEM with or without 500 mM sorbitol for 30 min. The cells were fixed in 3.7% paraformaldehyde. The coverslips were mounted using Vectashield (Vector Laboratories, Burlingame, CA, USA). Fluorescence imaging was performed using an Olympus FV1000-D (IX81) confocal laser scanning microscope (Olympus, Tokyo, Japan). Images were acquired using FV-10 ASW software (Olympus).

To observe stress granule markers, Ras GTPase-activating protein SH3-domain-binding protein (G3BP1) and T-cell intracellular antigen 1 related protein (TIAR), cells were fixed and then permeabilized in phosphate-buffered saline containing 0.1% Triton X-100 and 1% bovine serum albumin. Coverslips were incubated with an anti-G3BP1 (Cat. #: 611126, BD Biosciences, Franklin Lakes, NJ, USA) or anti-TIAR (Cat. #: 610352, BD Biosciences) mouse monoclonal antibody for 1 h and then incubated with Alexa 594-conjugated anti-mouse IgG (Molecular Probe) for 1 h.

### 2.4 Immunoprecipitation

COS-7 cells (100 mm dish) were lysed in 500 μL of HEPES buffer (50 mM, pH 7.2) containing 100 mM NaCl, 5 mM MgCl_2_, 1% Nonidet P-40 and cOmplete™ protease inhibitor cocktail (EDTA-free, Sigma-Aldrich). Cell lysates were subjected to immunoprecipitation with anti-FLAG monoclonal antibody (Cat. #: F1804, Sigma-Aldrich) and Protein A/G PLUS-agarose beads (Santa Cruz Biotechnology) as described previously [38,39]. The immunoprecipitates were boiled in SDS sample buffer.

### 2.5 Western blotting

COS-7 cell lysates (20 μg) and immunoprecipitates were separated by SDS-polyacrylamide gel electrophoresis. Western blotting was performed as previously described [40,41] using an anti-GFP (Cat. #: sc-9996, Santa Cruz Biotechnology, Santa Cruz, CA, USA), anti-FLAG or anti C-Raf (Cat. #: 610152, BD Biosciences) antibody along with a peroxidase-conjugated anti-mouse IgG (Jackson ImmunoResearch Laboratories, West Grove, PA, USA) antibody.

## 3 Results

### 3.1 DGKη selectively colocalizes with ASK3 in response to osmotic shock

**Fig. 1:**
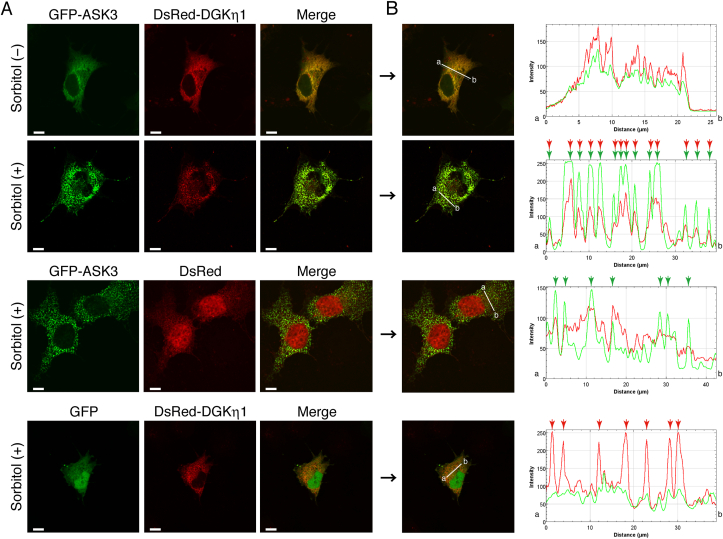
Subcellular localization of DGKη1 and ASK3 in COS-7 cells in response to osmotic shock. (A) Either pDsRed-monomer alone or pDsRed-monomer-DGKη1 was cotransfected with either pEGFP alone or pEGFP-ASK3 in COS-7 cells as indicated. After 24 h of transfection, the cells were incubated in the presence or absence of 500 mM sorbitol for 30 min. After osmotic shock, the cells were fixed and imaged. Representative data from three independent experiments are shown. Scale bars, 20 μm. (B) The distribution of DsRed-monomer-DGKη1 and EGFP-ASK3 were quantified using ImageJ software. Obvious punctate structures in the presence of 500 mM sorbitol are indicated with arrows. Green, EGFP; Red, DsRed-monomer. . (For interpretation of the references to colour in this figure legend, the reader is referred to the Web version of this article.)

We previously reported that DGKη translocated from the cytoplasm to punctate structures in an osmotic shock-dependent manner [13,27]. ASK3 is also known to act in response to osmotic stress [35,36]. Therefore, we first examined whether DGKη colocalizes with ASK3 in an osmotic shock-dependent fashion. For this purpose, we utilized DGKη1, which is a primary alternative splicing product of the DGKη gene [13,16]. When DsRed-monomer-tagged DGKη1 was coexpressed with EGFP-tagged ASK3 in COS-7 cells, they each showed cytoplasmic distribution and were partially colocalized (Fig. 1A). When the cells were exposed to osmotic shock (500 mM sorbitol for 30 min), DsRed-monomer-DGKη1 and EGFP-ASK3 were markedly and exclusively colocalized at punctate structures (Fig. 1A). Densitometric analysis of confocal microscopy exhibits that DGKη1 and ASK3 were almost perfectly colocalized at punctate structures in osmotic-shocked COS-7 cells (Fig. 1B). However, DsRed-monomer-DGKη1 did not colocalize with EGFP alone, even in the presence of osmotic stress (Fig. 1). Moreover, EGFP-ASK3 failed to codistribute with DsRed-monomer alone under the same conditions (Fig. 1).

**Fig. 2:**
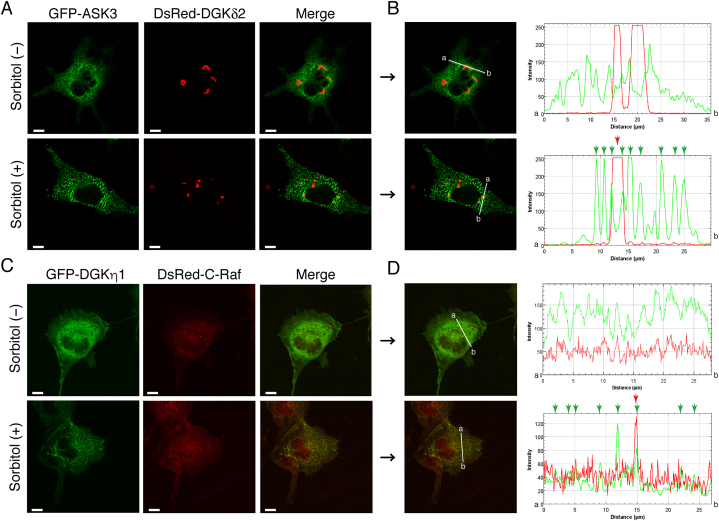
Subcellular localization of ASK3, DGKδ2, DGKη1 and C-Raf in COS-7 cells in response to osmotic shock. (A) pDsRed-monomer-DGKδ2 was cotransfected with pEGFP-ASK3 in COS-7 cells. (C) DsRed-monomer-C-Raf was cotransfected with pEGFP-DGKη1 in COS-7 cells. After 24 h of transfection, the cells were incubated in the presence or absence of 500 mM sorbitol for 30 min. After the osmotic shock, the cells were fixed and imaged. Representative data from three independent experiments are shown. Scale bars, 20 μm. (B, D) The distribution of DsRed-monomer-DGKδ2 and EGFP-ASK3 (B) and DsRed-monomer-C-Raf and EGFP-DGKη1 (D) was quantified using ImageJ software. Obvious punctate structures in the presence of 500 mM sorbitol are indicated with arrows. Green, EGFP; Red, DsRed-monomer. . (For interpretation of the references to colour in this figure legend, the reader is referred to the Web version of this article.)

DGKδ also belongs to the type II DGKs, and its structure is closely related to that of DGKη [42]. Thus, we determined next if DGKδ2, which is a main alternative splicing product of the DGKδ gene [43], is colocalized with ASK3. However, as shown in Fig. 2, DsRed-monomer-DGKδ2 was not colocalized with EGFP-ASK3, even in the presence of osmotic shock (Fig. 2).

We previously reported that DGKη1 was colocalized and interacted with C-Raf in an EGF stimulation-dependent manner in HeLa cells [26]. However, AcGFP-DGKη1 did not exhibit marked colocalization with DsRed-monomer-C-Raf, even under osmotic stress (Fig. 2). Collectively, these results indicate that DGKη1 was selectively colocalized with ASK3 in response to osmotic shock.

### 3.2 Punctate structures where DGKη and ASK3 are located are distinct from stress granules

**Fig. 3:**
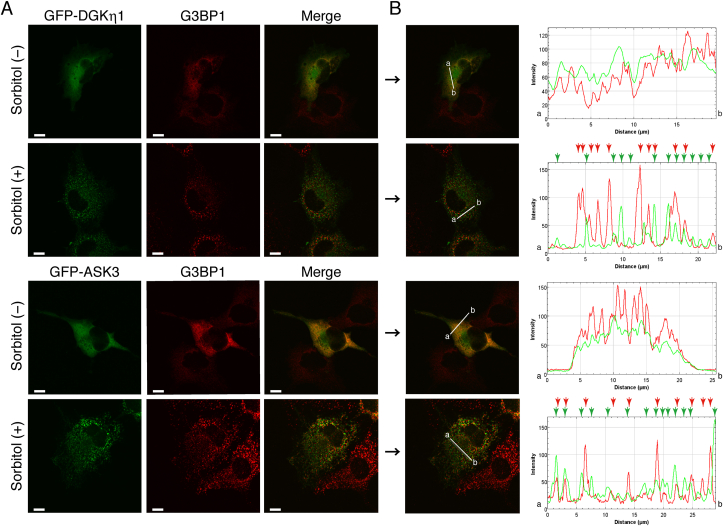
Subcellular localization of DGKη1, ASK3 and a stress granule marker (G3BP1) in COS-7 cells. (A) pEGFP-DGKη1 or pEGFP-ASK3 was transfected into COS-7 cells. After 24 h of transfection, the cells were incubated in the presence or absence of 500 mM sorbitol for 30 min and then were stained with mouse monoclonal anti-G3BP1 (stress granule marker) and Alexa Fluor 594-conjugated anti-mouse IgG antibodies. Representative data from three independent experiments are shown. Scale bars, 20 μm. (B) The distribution of EGFP-DGKη1, EGFP-ASK3 and G3BP1 was quantified using ImageJ software. Obvious punctate structures in the presence of 500 mM sorbitol are indicated with arrows. Green, EGFP; Red, DsRed-monomer. . (For interpretation of the references to colour in this figure legend, the reader is referred to the Web version of this article.)

Stress granules, which are dense aggregations composed of proteins and RNAs in the cytosol, appear when the cell is under stress, including osmotic shock [44]. Therefore, we investigated whether DGKη and ASK3 colocalize in stress granules. As shown in Fig. 3, Ras GTPase-activating protein SH3-domain-binding protein (G3BP1), a stress granule marker [45], was not located at punctate structures where DGKη1 existed. In addition to DGKη1, ASK3 also failed to codistribute with G3BP1. Moreover, another stress granule marker, T-cell intracellular antigen 1 related protein (TIAR) [46], also failed to colocalize with DGKη1 (Suppl. Fig. 1). These results strongly suggest that DGKη1 and ASK3 translocated from the cytoplasm to their specialized structures but not to general stress granules.

### 3.3 DGKη interacts with ASK3 in response to osmotic shock

**Fig. 4:**
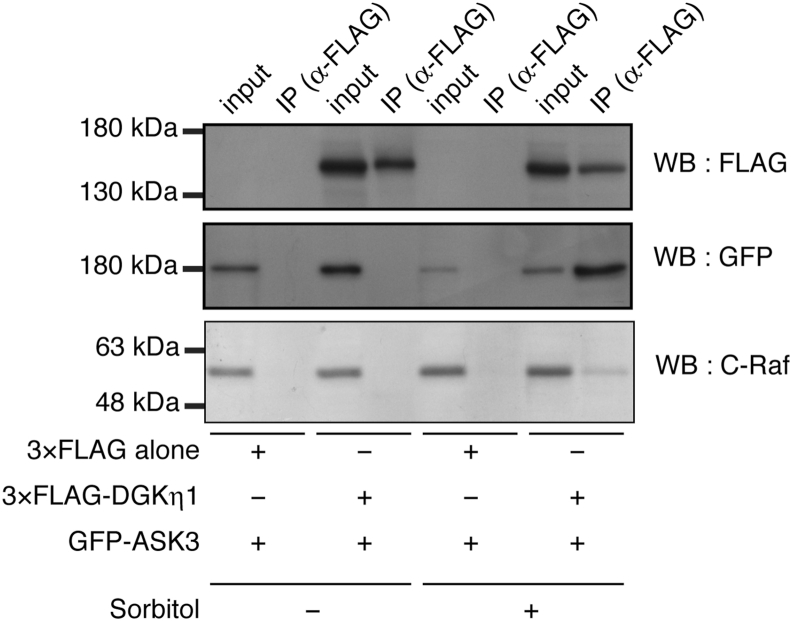
DGKη1 interacts with ASK3 in response to osmotic shock. pEGFP-tagged ASK3 was cotransfected with either p3 × FLAG vector alone or p3 × FLAG-tagged DGKη1 in COS-7 cells for 24 h and then treated with or without 500 mM sorbitol for 30 min. 3 × FLAG-DGKη1 was immunoprecipitated by the anti-FLAG antibody. Immunoprecipitated 3 × FLAG-DGKη1 and EGFP-ASK3 and endogenous C-Raf were analyzed by Western blotting with anti-FLAG, anti-GFP and anti-C-Raf antibodies, respectively. Representative data from three independent experiments are shown.

We next examined whether DGKη1 interacts with ASK3. To test this, we performed immunoprecipitation using COS-7 cells expressing 3 × FLAG-tagged DGKη1 and EGFP-tagged ASK3. EGFP-ASK3 was not detected in the immunoprecipitant of 3 × FLAG-DGKη1 using an anti-FLAG antibody in the absence of osmotic shock (Fig. 4). However, 3 × FLAG-DGKη1, but not 3 × FLAG alone, strongly coimmunoprecipitated with EGFP-ASK3 in the presence of osmotic stress (Fig. 4), indicating that DGKη1 interacts with ASK3 in an osmotic shock-dependent manner.

We previously reported that DGKη interacted with C-Raf in response to EGF [26]. Therefore, we determined next whether 3 × FLAG-tagged DGKη1 interacts with endogenous C-Raf in osmotic-shocked COS-7 cells. However, C-Raf was only slightly coimmunoprecipitated with 3 × FLAG-DGKη1 in response to osmotic shock (Fig. 4). These results indicated that DGKη1 is associated with C-Raf in addition to ASK3 under osmotic stress conditions.

Altogether, the results obtained in the present study indicate that DGKη1 and ASK3 selectively colocalize and associate with each other in specialized structures in response to osmotic stress.

## 4 Discussion

In the present study, we revealed for the first time the osmotic stress-dependent colocalization and interaction between DGKη1 and ASK3. The lipid kinase DGK metabolizes signal lipids, diacylglycerol and phosphatidic acid, which each regulate diverse physiological and pathological events [[1], [2], [3], [4], [5],^25^,^47^]. ASK3, which is a MAPKKK, controls bidirectional cell volume and blood pressure in response to hypo- and hyperosmotic shocks [35,36]. Although DGKη1 and ASK3 have been independently studied, it is interesting that these physiologically important enzymes colocalize and interact with each other under osmotic stress conditions (Fig. 1, Fig. 4), implying a functional linkage between them.

We found colocalization of DGKη1 with ASK3 at punctate structures in an osmotic shock-dependent manner (Fig. 1). Stress granules are dense aggregations in the cytosol and appear under stressful conditions, including osmotic shock [44]. However, the stress granule markers G3BP1 and TIAR failed to colocalize with DGKη1 and ASK3. Therefore, it is likely that the punctate structures where DGKη1 and ASK3 exist are not canonical stress granules, and consequently, are unique structures specialized for these enzymes that have not yet been identified. We previously showed that early endosome antigen 1 and clathrin partially colocalized with DGKη1 under osmotic stress [27]. Thus, the unknown structures may partly be a kind of clathrin-coated endosome-like vesicle. Recently, Watanabe et al. reported that ASK3 forms condensates, which are membraneless unlike the classic organelles surrounded with lipid bilayers, under hyperosmotic stress [48]. Therefore, it is possible that DGKη1 is also translocated to liquid-like condensates through the interaction with ASK3 in response to osmotic shock. Because DGKη (DGKη2) forms oligomers via its sterile α domain [13], DGKη may contribute to the formation of ASK3 condensates.

DGKη1 and ASK3 were distributed in the cytoplasm in unstimulated cells. However, they did not interact with each other in unstimulated cells (Fig. 4). Therefore, it is likely that DGKη1 and ASK3 are not markedly colocalized in resting cells. DGKη1 was located in punctate structures without ASK3 expression, and *vice versa* (Fig. 1). Therefore, it is likely that DGKη1 and ASK3 do not recruit each other.

DGKδ [42], a closely related isozyme of DGKη, did not distribute to the punctate structures where ASK3 and DGKη1 were localized, indicating that the subcellular localization of these isozymes is distinctly regulated. The subcellular localization of DGKδ was reported to be regulated by phosphorylation induced by phorbol 12-myristate 13-acetate and conventional protein kinase C [38,49].

DGKη1 interacted with C-Raf in response to EGF stimulation and associated with ASK3 under osmotic stress [26]. Both C-Raf and ASK3 are MAPKKKs. Thus, DGKη1 likely interacts with distinct MAPKKKs in differentially stimulated cells. ERK (MAPK) acts downstream of DGKη1-C-Raf (MAPKKK) in EGF-stimulated cells [26]. In contrast, JNK and P38 [35] function downstream of ASK3. ASK3 also regulates WNK1–SPAK/OSR1 signaling [36]. However, it remains unclear whether DGKη1 utilizes these pathways under osmotic stress.

The colocalization and interaction between DGKη1 and ASK3 (Fig. 1, Fig. 4) imply their functional linkage. Because ASK3 (NCBI, https://www.ncbi.nlm.nih.gov/gene/389840) and DGKη (NCBI, https://www.ncbi.nlm.nih.gov/gene/160851) are broadly expressed in many tissues, they can also work together in a broad range of tissues. The ASK3 gene is reportedly related to neurodegenerative diseases (Alzheimer's disease) [30]. Interestingly, genome wide association studies (GWAS Central, https://www.gwascentral.org) also suggested that DGKη, which is highly expressed in the brain [16], is associated with neurodegenerative diseases, such as Alzheimer's disease. In addition, DGKη is implicated in BPD [22,23]. The etiology of BPD is correlated with chronic stress [50]. Thus, stress may influence the pathogenesis of neurodegenerative diseases and BPD through DGKη and ASK3.

In the present study, for the first time, we identified the colocalization and interaction of DGKη and ASK3 in punctate granules that are specialized for DGKη and ASK3, in response to osmotic shock. The exclusive colocalization and interaction of DGKη and ASK3 imply their functional linkage, although it needs to be explored further. It will be interesting to determine the identity of the structures and the functional linkage between DGKη and ASK3.

## Declaration of competing interest

The authors declare no conflicts of interest associated with the content of this article.
