# Supplementary material for: Diacylglycerol kinase η colocalizes and interacts with apoptosis signal-regulating kinase 3 in response to osmotic shock
Source: Biochem Biophys Rep. 2021 Apr 27;26:101006. doi: 10.1016/j.bbrep.2021.101006 (PMC8100535; doi:10.1016/j.bbrep.2021.101006)
Supplement: Multimedia component 1 [file mmc1.pdf]

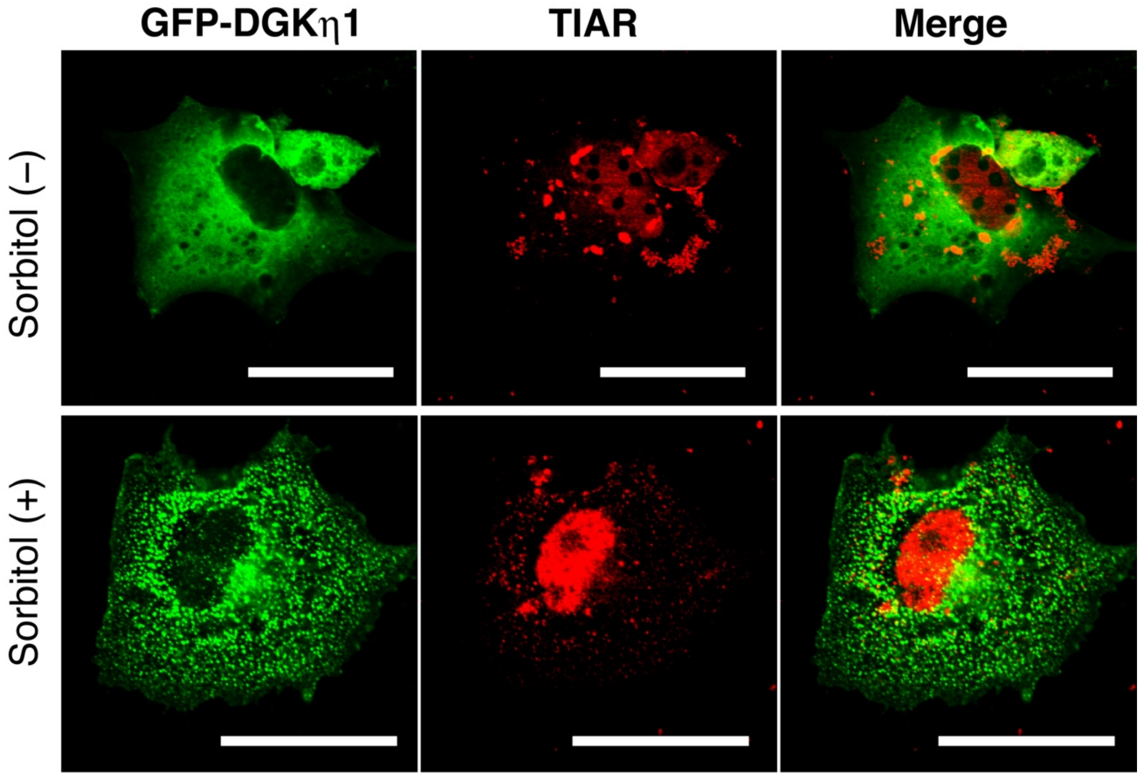

Suppl. Fig. 1. Subcellular localization of DGKη1 and a stress granule marker (TIAR) in COS-7 cells

(A) pAcGFP-DGKη1 was transfected into COS-7 cells. After 24 h of transfection, the cells were incubated in the presence or absence of 500 mM sorbitol for 30 min and then were stained with mouse monoclonal anti-TIAR (stress granule marker) and Alexa Fluor 594-conjugated anti-mouse IgG antibodies. Representative data from three independent experiments are shown. Scale bars, 30 μm.
